# 食管癌术后纵隔感染不常见，但更重要

**DOI:** 10.3779/j.issn.1009-3419.2018.04.28

**Published:** 2018-04-20

**Authors:** 俊峰 刘

**Affiliations:** 河北医科大学第四医院胸外科 Department of Thoracic Surgery, Fourth Hospital Affiliated to Hebei Medical University

食管癌术后纵隔感染并不少见，但大部分患者，感染物能自行引流入胸腔，通过胸腔引流治愈，而不会引起严重后果。但在少数情况下，纵隔感染与胸腔隔离，造成引流困难，如果处理不及时，可出现明显症状，甚至造成严重并发症。

食管癌术后纵隔感染的原因不外乎术中污染，如无菌意识不强造成消化液外溢、切除的过程中造成肿瘤破裂、对于纵隔污染区域没有进行彻底冲洗和消毒。纵隔内植入物如止血材料等感染和消化道漏也是造成严重纵隔感染的原因。

前已述及，大部分纵隔感染可以引流至胸腔，对于此类纵隔感染，患者症状一般很轻，如发烧、寒战、白细胞升高等，经胸腔闭式引流、抗生素应用以及营养支持等治疗后会逐渐恢复。少部分患者感染积聚于纵隔，不能自行引流至胸腔，除出现感染中毒症状以外，还可以出现气管压迫症状，造成呼吸困难，甚至造成气管（支气管膜）部穿孔或穿通大血管，危及患者生命。

CT检查对于自行引流至胸腔的纵隔感染，纵膈内一般不会有阳性发现，仅表现为不同程度的胸腔积液。CT检查对于局限于纵隔内感染的诊断有重要价值。CT上表现为纵隔内含有液性区域，可以压迫气管膜部。如果为消化道漏所引起，消化道造影可见造影剂流入纵隔，并会出现液气平面。根据CT检查，可以确定感染积液的部位，对于决定处理方法非常重要。

彻底引流是纵隔感染处理的首选原则，对于自行引流至胸腔的纵隔感染，彻底引流胸腔通常会产生很好的效果。最棘手的是感染局限于纵隔，会给引流造成严重困难。对于积聚于上纵隔的感染，经颈部纵隔引流是很好的选择，特别是经颈部进行食管胃吻合的患者，可以打开原颈部切口，将引流管经胸廓入口下至上纵隔进行引流。对于脓液积聚于隆突下的患者，特别是有气管压迫症状的患者，可以在支气管镜监视下，经气管膜部穿刺引流，同时根据药敏结果，注入抗生素抗感染治疗。对于食管胃吻合口漏引起的纵隔感染，且不能引流至胸腔的，可以在X线透视监视下，将细引流管经漏口导入纵隔，进行所谓“内引流”，待感染控制后再逐渐拔除引流管。对于上述方法不便于处理的或处理后效果不佳的严重纵隔感染，可采用电视胸腔镜或电视纵隔镜手术，清除纵隔内感染和坏死组织，清洗纵隔和胸腔，彻底引流纵隔和胸腔^[1]^。

个别纵隔感染会穿破气管、主动脉，甚至消化道（也可以是消化道漏引起纵隔感染而穿入气管，引起消化道——气道漏），危机患者生命。根据我们的经验，纵隔感染穿破气管，呼吸功能难以维持的，应积极进行手术引流和气管修补，术中应当将胸腔与纵隔的分隔彻底分开以便胸腔引流，同时去除感染的坏死组织，彻底清洗和消毒纵隔，修补气管（支气管）漏口，必要时用带蒂肌瓣加强修补部位，最后放置胸腔与纵隔引流管。尽管漏口有不能一期愈合的可能，但此处理使术野很快粘连，即便两周后气管漏再次发生，因其它部位已经粘连，气体会通过纵隔引流管流出，不会对呼吸功能造成严重影响，随着肉芽组织的增生，漏口会逐渐愈合。若纵隔感染造成食管气管漏发生，大部分患者不会对呼吸和循环造成严重影响，通过导入十二指肠营养维持营养等治疗，大部分患者漏口可愈合，少部分成为慢性食管气管漏。个别患者食管气管漏一旦发生，呼吸功能难以维持，对于此类患者，进行食管或气管支架治疗多因并发症死亡，不是正确选择，再次手术治疗可给患者提供生存机会。术中应进行气管漏口修补，必要时带蒂肌瓣加强。对于消化道漏口可根据情况采取切除再吻合、漏口修补等。纵隔感染的另一严重并发症就是大动脉漏或大动脉消化道漏，对于此并发症，进行大动脉漏修补无成功的报告^[2]^，目前唯一可行的办法就是安放主动脉支架。

总之，食管癌术后纵隔感染的处理应根据病变的部位和严重程度选择不同的防范进行引流，尽可能采取创伤较小的方法，达到最好的引流效果，对出现严重并发症的纵隔感染，要及时恰当处理。
